# Bone histomorphometric changes in children with rheumatic disorders on chronic glucocorticoids

**DOI:** 10.1186/s12969-016-0119-z

**Published:** 2016-11-10

**Authors:** Jennifer Harrington, Douglas Holmyard, Earl Silverman, Etienne Sochett, Marc Grynpas

**Affiliations:** 1 Department of Pediatrics, Hospital for Sick Children, University of Toronto, Toronto, Canada; 2 Department of Pathology and Laboratory Medicine, Mount Sinai Hospital, Toronto, Canada; 3 Samuel Lunenfeld Research Institute, Mount Sinai Hospital, University of Toronto, Toronto, Canada; 4 Division of Endocrinology, Hospital for Sick Children, 555 University Avenue, Toronto, ON M5G1X8 Canada; 5 Division of Rheumatology, Hospital for Sick Children, Toronto, Canada

**Keywords:** Glucocorticoids, Juvenile idiopathic arthritis, Systemic lupus erythematosis, Osteoporosis, Bone histomorphometry, Bone mineralization

## Abstract

**Background:**

Rheumatic diseases are associated with an increased fracture risk. The tissue level characteristics of the bone involvement in children have not been well elucidated. Our objectives were to describe the bone micro-architectural characteristics in children with rheumatic diseases on chronic glucocorticoids, and to determine associations between micro-architectural findings with clinical and radiological variables.

**Methods:**

Children on chronic glucocorticoids for an underlying rheumatic disease were referred for evaluation of bone fragility given the presence of vertebral compression fractures. A trans-iliac bone biopsy was performed as part of the clinical assessment. Histomorphometric analysis and quantitative backscattered electron imaging (qBSE) of the biopsy samples were undertaken.

**Results:**

Data of 15 children (14.0 ± 3.2 years) with a duration of glucocorticoid exposure of 6.2 ± 4.1 years and average prednisone dose of 14.1 ± 6.2 mg/m^2^/day were assessed. Histomorphometric analyses demonstrated significant decrease in trabecular thickness (*p* = 0.01), osteoid thickness (*p* < 0.01), osteoblast surface (*p* = 0.02) and increase in trabecular separation (*p* = 0.04) compared to published age-matched normative data. Severity of the trabecular deficit was correlated to glucocorticoid dose, height and body mass index Z score, but not bone mineral density or measures of disease activity. Using qBSE to measure bone mineralization, the subjects were shown to have a heterogeneous and hypermineralized profile, with higher cumulative glucocorticoid dose being associated with greater mineralization (*p* < 0.01).

**Conclusions:**

In children with rheumatic diseases presenting with vertebral fractures, there is evidence of abnormal bone matrix mineralization and impairments of bone micro-architecture that correlate to glucocorticoid dose.

## Background

Children and adolescents with rheumatic diseases, such as systemic lupus erythematosis (SLE) and juvenile idiopathic arthritis (JIA) are recognized to be at risk for impaired bone health. In those who develop overt osteoporosis, multiple risk factors are implicated, including increased concentrations of inflammatory cytokines, diminished nutrition, physical activity and muscle bulk [1, 2]. Use of glucocorticoids, in particular, is also known to compromise bone mass, quality and strength [3]. As a consequence, in children with rheumatic conditions, a 10 to 30 % prevalence rate of vertebral fractures has been reported [4–7], with a 6 % incidence of vertebral fractures within 12 months of glucocorticoid initiation [8].

Bone mineral density (BMD) as measured by dual-energy x-ray absorptiometry (DXA), is one of the most common tools used to assess bone health. Low BMD is associated with an increased risk for fractures in otherwise healthy children [9]. Children with rheumatic diseases including SLE and JIA have been shown to have reduced BMD compared to healthy age-matched controls [1, 10–12]. Decreased lumbar spine BMD Z score in the first 6 months of initiation of glucocorticoid treatment has been demonstrated to be an important predictor for increased vertebral fracture risk [13]. DXA has, however, limitations in the assessment of bone health in pediatric cohorts given that the results are influenced by bone size [14]. In addition bone strength is determined not only by bone density but also by the underlying bone quality and micro-architecture.

Histomorphometric analysis of trans-iliac bone samples allows characterization of bone microarchitecture including bone quantity, structure and metabolism. Histomorphometric studies in adults with rheumatoid arthritis have shown evidence of reduced trabecular bone volume with decreased trabecular thickness and bone turnover [15, 16]. Moreover, in adult cohorts, vertebral fractures have been demonstrated to relate to trabecular bone micro-architecture independent of the bone density [17].

The mineral content of bone has similarly been demonstrated to be an important determinant of bone stiffness and strength [18]. The degree and heterogeneity of bone mineralization can be measured from trans-iliac bone samples using quantitative backscattered electron imaging (qBSE). Deviations in bone mineralization have been demonstrated in patients with bone fragility, such as in children with osteogenesis imperfecta [19] and adults with postmenopausal osteoporosis [20], but have not been assessed to date in patients with rheumatic diseases.

Studies of bone histomorphometry and mineralization in pediatric patients treated with glucocorticoids have contributed to the understanding of underlying pathology and clinical factors with the potential to predict severity of bone disease [21, 22]. Given these features have not to date been described in pediatric rheumatalogical patients, the aims of this pilot study were therefore to characterize bone histomorphometry and mineralization in a small cohort of children with rheumatic diseases and to assess associations between clinical and radiological indices to bone micro-architecture.

## Methods

All patients with a rheumatic disease who had been referred to the Bone Health Clinic at the Hospital for Sick Children, Toronto between 2003 and 2011 and who had undergone a trans-iliac bone biopsy were included in this retrospective descriptive analysis. Patients had been referred to the clinic for assessment of bone health because of the finding of at least one vertebral compression fracture. All patients were on calcium and vitamin D supplements and had undergone baseline investigations including biochemical assessment of parameters of calcium homeostasis, as well as spinal radiographs and DXA. The bone biopsy was undertaken as part of the clinical evaluation for symptomatic osteoporosis. Exclusion criteria included children with impaired renal function or other chronic medical conditions (other than their rheumatic disease) that could affect bone health. This study was approved by the Hospital for Sick Children’s Research Ethics Committee.

### Clinical data

Information was collected from the patients’ records regarding their underlying rheumatic disease, including age of diagnosis, duration of symptoms and treatment. All children were on glucocorticoid treatment (prednisone) at the time of the bone biopsy. Duration of glucocorticoid treatment, as well as cumulative and average daily dose was documented. All glucocorticoid doses were expressed as milligrams per body surface area (mg/m^2^) in prednisone equivalents. Clinical data including age, height and weight were collected from the time of the trans-iliac bone biopsy. Height was measured with a wall-mounted stadiometer to the nearest 0.1 cm and weight was taken on an electric digital scale to the nearest 0.1 kg. Height, weight and body mass index (BMI) [weight (kg)/height (m)^2^] z-score were calculated using reference data provided by the Centre for Disease Control 2000 standardized reference charts. Pubertal development was assessed according to Tanner criteria [23, 24]. As a measure of functional status of the subjects at the time of the bone biopsy, the Childhood Health Assessment Questionnaire (CHAQ) score was recorded. The CHAQ score is a validated, reliable tool for measuring functional status in children with juvenile idiopathic arthritis [25], and has also been demonstrated to reflect disease activity in other rheumatological conditions [26, 27]. CHAQ scores range from 0 (indicating no disability) to 3 (severe disability).

### Biochemistry

Biochemical markers taken at the time of the biopsy were recorded. Serum calcium, phosphate and alkaline phosphatase were measured by spectophotometry using the VITROS 950 Chemistry System (Johnson and Johnson Ortho-Clinical Diagnostics, Rochester, NY). Serum intact parathyroid hormone (PTH) was measured by IMMULITE chemoiluminescent immunometric assay (Siemens Healthcare Diagnostics, Deerfield, IL). Serum 25 hydroxyvitamin D (25(OH)D) was measured by high-performance liquid chromatography tandem mass spectroscopy or radioimmunoassay (DiaSorin S,p,A, Saluggia, Italy). Erythrocyte sedimentation rate (ESR) was used as a biochemical marker of rheumatological disease activity.

### Radiological studies

Lumbar spine (L1 to L4) areal BMD was measured by dual-energy X-ray absorptiometry (DXA) in the anterior-posterior direction (Lunar Prodigy; General Electric; Madison, WI, USA). Compressed lumbar vertebrae were excluded from the analysis. To account for bone size and height, bone mineral apparent density (BMAD) was derived using the method proposed by Kroger et al., where by BMAD = BMD × [4/(π x width)] [28]. Areal BMD and BMAD values were transformed into Z scores using the equipment specific age and sex-adjusted Canadian reference values for areal BMD and published reference data for BMAD [29].

Standard lateral thoracic and lumbar spine radiographs were obtained in an upright position. All radiographs were assessed for vertebral morphology and loss of vertebral height. Morphology was determined using the Genant semi-quantitative method by measuring the posterior, mid-body and anterior vertebral body height ratios as described previously [30]. Significant vertebral compression was defined as a height loss of greater than 20 %. Vertebral bodies with evidence of compression were assigned a severity score; grade 1 = mild (loss of height of > 20 to 25 %), grade 2 = moderate (loss of height of > 25 to 40 %), and grade 3 = severe (loss of height of > 40 %) [30].

### Trans-iliac histomorphometry

Utilizing ultrasound and fluoroscopic guidance, full thickness transiliac bone biopsies were obtained with a Rochester biopsy device under general anaesthetic from a site located 2 cm below and behind the anterior superior iliac spine for static histomorphometry. Informed consent was obtained from all subjects. Biopsy preparation and static histomorphometric analyses were performed as previously described [31]. In brief, the specimens were placed in 70 % ethanol, then dehydrated and embedded in polymethylmethacrylate. Histomorphometric parameters were evaluated in undecalcified sections treated with Goldner’s trichrome or Von Kossa stain. The total trabecular area was analyzed as serial fields using the Lietz Bioquant morphometry system (Bioquant Nova Prime, Version 6.50.10). Bone histology was reported using the terminology established by the Nomenclature Committee of the American Society of Bone and Mineral research [32]. Results were converted to percentage scores compared to published reference age and gender matched controls [31].

### Bone mineralization

The embedded iliac crest biopsy blocks were used for evaluation of bone mineralization density distribution (BMDD) using quantitative backscattered electron imaging (qBSE). The blocks were cut, polished, carbon-coated and imaged using backscattered electron imaging (solid state BSE detector, FEI Company, Hillsboro, OR, USA) on a FEI XL30 ESEM. This technique allows quantification of the intensity of the backscattered electron signal from the surface of a sectioned bone by calibration of the brightness and contrast. Histograms of the grey level distribution were created for the trabecular bone of the iliac crest samples, with a range of grey levels from 0 to 255. Increasing brightness corresponds with increasing mineralization [33]. The signal is proportional to the weight fraction of calcium in the embedded bone tissue. Two parameters were derived for statistical analysis; mineralization peak, the peak position on the histogram which indicates the most frequently occurring grey level in the bone area, and the full width at half-maximum height (FWHMH) of the bone mineralization histogram curve which describes the mineral heterogeneity of the sample.

### Statistical analysis

The data were analyzed using SPSS 21.0 for windows (SPSS Inc, Chicago, IL, USA). Anthropometry and densitometry Z scores were tested for significant deviation from zero using the one-sample *t*-test. Differences in histomorphometric measurements between the cohort and published normative data were assessed by comparing the subject’s results as expressed as a percentage of the reference mean versus 100 % using one-sample t-tests. Correlations between histomorphometric, bone mineralization and clinical variables were evaluated with Pearson and Spearman rank correlations. No adjustment for multiple comparisons was made. Statistical significance was inferred with a *P* value less than 0.05.

## Results

### Cohort clinical characteristics

Fifteen children (12 females) with a mean age of 14.0 ± 3.2 years were included in this analysis. Eight of the children had a diagnosis of SLE, 5 systemic JIA and 2 had a systemic vasculitis. The clinical characteristics of the cohort are displayed in Table 1. All patients had been commenced on glucocorticoid treatment within 2 months of clinical diagnosis.

**Table 1:** Clinical characteristics of the study cohort

Clinical variable	Results (*n* = 15)
Age at time of bone biopsy (years)	14.0 ± 3.2
Age at diagnosis of rheumatologic disease (years)	7.6 ± 3.6
Anthropometry at time of bone biopsy	
Height Z score	−2.3 ± 1.4
Weight Z score	−0.2 ± 1.4
Body mass index Z score	0.8 ± 1.2
Pubertal stage (% of subjects pre-pubertal: mid-pubertal: post-pubertal)^b^	33:40:27
Rheumatic condition characteristics	
Functional ability at time of bone biopsy (CHAQ score)	0.25 (0 to 1.87)^a^
Erythrocyte sedimentation rate (mm/h)	13 (1 to 57)^a^
Glucocorticoid treatment	
Duration of glucocorticoid treatment (years)	6.2 ± 4.1
Cumulative glucocorticoid dose (mg/m^2^)^c^	29134 ± 29410
Average glucocorticoid dose (mg/m^2^/day)^c^	14.1 ± 6.2
Number of vertebral fractures (number of subjects)	
1 vertebral fracture	5 (33 %)
2 to 4 vertebral fractures	3 (20 %)
≥ 5 vertebral fractures	7 (47 %)
Fracture severity (number of subjects)^d^	
Grade 1	3 (20 %)
Grade 2	10 (67 %)
Grade 3	2 (13 %)
Lumbar spine areal BMD Z score	−3.4 (−5.5 to −1.7)^a^
Lumbar spine BMAD Z score	−2.9 (−4.1 to −0.9)^a^
Serum 25(OH)D (nmol/L)	69.5 (43 to 118)^a^

Results are expressed as mean ± standard deviation unless otherwise indicated

^a^median (range)

^b^pre-pubertal: Tanner stage 1, mid-pubertal: Tanner stage 2 and 3, post-pubertal: Tanner stage 4 and 5

^c^reported in prednisone equivalents

^d^patients with multiple vertebral fractures were graded according to the highest severity score

Serum concentrations of calcium, phosphate, parathyroid hormone and creatinine were within the normal range for all subjects at time of the biopsy. The median 25(OH)D concentration was 69.5 (43 to 118) nmol/L, and only one patient had a concentration less than 50 nmol/L. Mean alkaline phosphatase was 133.9 ± 48.2 IU/L, with 6 patients having concentrations below the normal reference range for age and gender. ESR was mildly raised in most subjects with a median concentration of 13 (range 1 to 57) mm/h.

### Radiological measures of bone health

Twelve of the fifteen patients were investigated for vertebral compression fractures due to the onset of back pain, while the remaining three had an X-ray of their spine performed following the finding of a lumbar BMD Z score less than −2 on DXA. There were a total of 68 vertebral fractures within the entire cohort, with the majority involving the thoracic region (71 %). Almost 50 % of the cohort had fractures involving 5 or more of the vertebrae, with over half of the subjects having grade 2 severity vertebral height loss (loss of height of > 25 to 40 %) (Table 1). Greater number of vertebral compression fractures was associated with higher BMI Z score (*r* = 0.78, *p* < 0.01). None of the patients had a history of peripheral fractures. Greater severity of vertebral height loss was associated with higher cumulative glucocorticoid dose (*r* = 0.56, *p* = 0.03), longer disease duration (*r* = 0.60, *p* = 0.02) and higher CHAQ score (*r* = 0.60, *p* = 0.02).

The lumbar spine areal BMD and BMAD were significantly below results expected in healthy subjects of the same age and gender (*p* < 0.001). There was, however, a spectrum of values, and 6 of the 15 of the subjects had a lumbar spine BMAD Z score greater than −2.0. Lower lumbar spine BMAD Z score was associated with greater average glucocorticoid dose (*r* = −0.50, *p* = 0.04), but not cumulative dose or duration of glucocorticoids. There was no significant association between number or severity of vertebral compression factures and BMD.

### Trabecular iliac bone histomorphometry

Iliac bone histomorphometry demonstrated evidence of thinning of the trabecular with increased separation, although preservation of the trabecular number (Table 2). Bone formation activity was decreased with low markers of osteoid formation and percentage of osteoblast-covered surface. There was no significant difference in histomorphometric values in the subjects with JIA compared to those with SLE.

**Table 2:** Static histomorphometric results from trans-iliac bone biopsies

	Raw results	% Reference mean^a^	*p* value^b^
Structural parameters			
Bone volume (BV/TV) (%)	20.9 ± 7.0	82.9 ± 28.9	0.06
Trabecular number (Tb. N) (#/mm)	1.78 ± 0.4	92.8 ± 36.1	0.54
Trabecular thickness (Tb. Th) (μm)	107 ± 21	76.0 ± 22.1	<0.01
Trabecular separation (Tb. Sp) (μm)	505 ± 101	115.9 ± 21.3	0.04
Formation parameters			
Osteoid surface (OS/BS) (%)	16.5 ± 3.7	83.0 ± 16.2	0.01
Osteoid volume (OV/BV) (%)	1.8 ± 0.6	88.8 ± 18.7	0.06
Osteoid thickness (O.Th) (μm)	4.6 ± 0.6	71.9 ± 18.6	<0.01
Osteoblast surface (Ob.S/BS) (%)	5.0 ± 2.7	62.8 ± 21.0	<0.01

All data is presented as mean ± standard deviation

^a^Average of percentage of age-matched reference mean published previously

^b^Histomorphometry results (structural and formation parameters) were converted to percentiles compared to published age matched normative data prior to analysis

Univariate analysis showed that higher average glucocorticoid dose was associated with greater deficits in both bone structural and formation parameters. (Table 3). In particular, the amount of osteoblast surface per bone surface percentage strongly associated with both dose and duration of glucocorticoids. Systemic clinical effects of glucocorticoids (higher BMI and lower height Z score), also correlated with histomorphometric parameters. Lower bone volume and osteoblast surface associated with a greater number of vertebral compression fractures. There was no association between bone histomorphometry with lumbar spine areal BMD or BMAD Z scores, 25(OH)D or ESR concentrations.

**Table 3:** Clinical correlates with trans-iliac histomorphometry

Clinical variable	Structural parameters		Formation parameters	
	*Bone volume (BV/TV)* ^a^	*Trabecular Thickness* *(Tb. Sp)* ^a^	*Osteoid thickness* *(O.Th)* ^a^	*Osteoblast surface (Ob.S/BS)* ^a^
Average glucocorticoid dose (mg/m^2^/day)	**−0.61 (** ***p*** **= 0.01)**	**−0.66 (** ***p*** **< 0.01)**	**−0.61 (** ***p*** **= 0.01)**	**−0.88 (** ***p*** **< 0.01)**
Cumulative glucocorticoid dose (mg/m^2^)	−0.32 (*p* = NS)	−0.25 (*p* = NS)	−0.39 (*p* = NS)	**−0.89 (** ***p*** **< 0.01)**
Glucocorticoid duration	−0.11 (*p* = NS)	−0.17 (*p* = NS)	−0.30 (*p* = NS)	**−0.52 (** ***p*** **= 0.04)**
Height Z score	0.35 (*p* = NS)	**0.50 (** ***p*** **= 0.04)**	0.43 (*p* = NS)	**0.70 (** ***p*** **< 0.01)**
BMI Z score	−0.33 (*p* = NS)	−0.29 (*p* = NS)	−0.19 (*p* = NS)	**−0.70 (** ***p*** **< 0.01)**
Number of vertebral compression fractures	**−0.51 (** ***p*** **= 0.04)**	−0.23 (*p* = NS)	−0.43 (*p* = NS)	**−0.87 (** ***p*** **< 0.01)**
Lumbar spine areal BMD Z score	0.19 (*p* = NS)	0.26 (*p* = NS)	0.20 (*p* = NS)	0.40 (*p* = NS)
Lumbar spine BMAD Z score	0.13 (*p* = NS)	0.05 (*p* = NS)	−0.10 (*p* = NS)	0.25 (*p* = NS)

Data are Pearson’s correlation r (*p* value). ﻿﻿All significant results (*p* < 0.05) are highlighted in bold﻿. ﻿NS = not significant

^a^Histomorphometry results were converted to percentiles compared to published age matched normative data prior to analysis

The rheumatological patients had a mineralization peak of 167.4 ± 20.5 and a FWHMH of 34.3 ± 7.2. The bone mineralization of the patient cohort was compared to published normative values for healthy children [34]. The rheumatological patients had a shift in the mineral distribution towards a more hypermineralized profile (*p* <0.001) and had a greater heterogeneity in the mineralization distribution (*p* < 0.01). Greater cumulative glucocorticoid dose (*r* = 0.71, *p* < 0.01), lower histomorphometric bone formation parameters (*r* = −0.7, *p* < 0.01) and lower serum alkaline phosphatase concentration were associated with a more hypermineralized profile (*r* = −0.52, *p* = 0.04). Bone mineralization variables did not related to age, anthropometry or lumbar spine bone mineral density.

## Discussion

This study demonstrated that children with rheumatic diseases on chronic glucocorticoids who presented with vertebral fractures, have evidence of significantly decreased bone structural and formation histomorphometric indices compared to healthy age matched peers. The bone mineralization findings revealed greater heterogeneity of mineralization as well as a significant shift towards hypermineralization within the trabecular bone.

Patients with rheumatic conditions are at increased risk for bone fragility and fractures. [5–7]. Higher glucocorticoid dose have been shown to correlate with the presence of vertebral fractures [7, 8]. Similarly in our study, those children with greater cumulative glucocorticoid dose had a more severe grade of vertebral height loss. The results from this study add to this literature by demonstrating that the underlying histomorphometric deficits in trabecular bone in these children also relate to glucocorticoid dose. In our cohort, structural and formation parameters were lower than the age-specific reference range. Adults with rheumatoid arthritis have been shown to have evidence of decreased trabecular bone volume and turnover [15, 35]. Reduced trabecular thickness and osteoblast surface have similarly been found in other cohorts of children treated with glucocorticoids such as those with Duchene muscular dystrophy and steroid dependent nephrotic syndrome [21, 22]. Unlike, however, the histomorphometry results from the pediatric patients with nephrotic syndrome, our subjects did not have evidence of osteomalacia, as seen by the reduced osteoid thickness and volume. The primary effect of the glucocorticoid treatment in our subjects would appear to be through the reduction in bone formation decreasing osteoblastogenesis, increasing osteoblast apoptosis and inhibiting osteoblast production of bone matrix [36–38].

As well as the relationship between glucocorticoid dose and trabecular bone deficit, we were able to demonstrate that those children with more pronounced systemic effects from their steroid treatment (specifically reduced height and increased BMI Z score) have lower trabecular thickness and osteoblast surface. This association between increased BMI and greater bone fragility has also been demonstrated in a prospective study of children with rheumatic conditions which assessed determinants of vertebral fractures [13]. Combined, these findings would support that clinicians should have heightened vigilance around bone health screening in those rheumatological patients with systemic glucocorticoid effects.

In addition to structural and formation deficits, children with rheumatic diseases on glucocorticoids have significant differences in trabecular bone mineralization compared to healthy controls. Trabecular bone consists of individual osteons and bone packets that have different mineral contents depending on when they were produced during the (re) modeling cycle. During secondary mineralization the mineral content of these bone packets increases with time as free water in the bone matrix is replaced by mineral [18]. Bone mineralization as measured by qBSE quantifies the degree and heterogeneity of mineralization across the cross-sectional area of the trans-iliac bone biopsy, and is determined by bone turnover rate and by the time course of mineral accumulation within the bone matrix [18]. In our subjects, the shift in the bone mineralization towards a more hypermineralized profile would be consistent with decreased bone turnover leading to a greater percentage of mineralized bone packets. The inverse association between mineralization peak and histomorphometric bone formation parameters as well as serum bone turnover marker alkaline phosphatase, would support this finding. The increased heterogeneity of the matrix mineralization is also likely a result of a significant decrease in bone turnover. In experimental models, decreased bone turnover has been shown to lead to increased heterogeneity in bone mineralization [39]. Increased degree of mineralization through decreased bone remodeling is probably a significant contributor to the underlying bone fragility in our subjects. In other conditions, bone hypermineralization has been demonstrated to contribute to bone brittleness and tendency to fracture [40, 41].

BMD as measured by DXA is a commonly used screening tool to assess bone health in children on chronic glucocorticoids. A meta-analysis demonstrated a difference of −0.18 [−0.25; −0.1] g/m^2^ lower spine BMD in children with JIA on glucocorticoids compared to healthy controls [42]. Similarly we demonstrated an association between average daily glucocorticoid dose and BMAD Z score. While low bone mass in pediatric patients has been defined by a BMD Z score of less than or equal to −2 [43], only 60 % of our cohort, all of whom had vertebral compression fractures, had a lumbar spine BMAD Z score that met this criteria. We also did not find a correlation of either lumbar spine areal BMD or BMAD to the histomorphometric or mineralization indices. While the density of bone is measured in BMD values, our findings would suggest that DXA BMD values do not always reflect some of the other underlying pathological mechanisms that glucocorticoids have on bone. In addition clinical variables such as short stature, delayed skeletal maturation, ethnicity and lean body mass can confound BMD results [43, 44]. The lack of association of bone histomorphometry to BMD highlights the limitations of relying on BMD values alone to evaluate bone health in children with chronic diseases. Given its invasive nature, trans-iliac bone biopsies are not suitable as a bone health screening tool, but should be limited to those patients who appear to have severe bone fragility out of keeping of their medical history or bone health risk factors. Instead, yearly lateral thoracolumbar spine X-rays to assess for vertebral height loss should be used in conjunction with BMD values to screen children with rheumatic conditions on glucocorticoids for evidence of bone fragility.

The treatment of bone fragility in children with rheumatic conditions is challenging, in part because of limited therapeutic options in the pediatric age range. Given higher rates of vitamin D deficiency in children with rheumatic conditions [45, 46], ensuring adequate vitamin D and calcium intake to maintain bone mineralization is an important first line therapy, although may not be sufficient to help prevent fragility fractures [13]. The use of biologic agents in JIA, has been demonstrated to help facilitate new bone formation after discontinuation of glucocorticoids [47]. These effects are however, attenuated in those patients who remain on glucocorticoids [48]. Bisphosphonates have been shown to improve BMD in children with secondary osteoporosis [49, 50]. Our findings of low baseline osteoblast activity in children on chronic glucocorticoids, emphasizes the need for cautious use of anti-resorptive agents such as bisphosphonates, which can lead to further suppression of bone turnover [21]. Consideration of commencing bisphosphonate therapy should be reserved for those patients presenting with significant fragility fractures or bone pain.

There are limitations to our study that deserve consideration, in particular the small number of subjects included in this analysis. Given the small subject numbers further data from a larger number of subjects is needed to confirm the findings from this pilot study. In addition the subjects in our study presented with a significant degree of bone fragility and vertebral compression fractures. It is uncertain to what degree similar histomorphological and mineralization deficits would be seen in a cohort of asymptomatic children on equivalent glucocorticoid doses. While we demonstrated a linear relationship between glucocorticoid dose and trabecular structural deficits, we cannot comment as to whether there is a threshold glucocorticoid dose below which there would not be a significant detrimental effect on bone quality. Only static and not dynamic histomorphometry was able to be performed, as the subjects had not received tetracycline prior to the biopsy being taken.

## Conclusions

In conclusion, our results from this small pilot study demonstrate that children with rheumatic diseases on chronic glucocorticoid treatment who present with vertebral compression fractures have evidence of decreased trabecular bone volume and formation with increased and more heterogeneous mineralization. The association between glucocorticoid dose to the bone micro-architectural deficits, highlights the importance of decreasing the steroid dose in chronic disease states where possible. Treatment with bisphosphonates is sometimes used in children with glucocorticoid induced osteoporosis, however can lead to further suppression of bone formation [21]. Our findings support the need for further research in anabolic treatment options for children, in order to address the underlying bone pathology.

## Abbreviations

25(OH)D: 25 hydroxyvitamin D
BMAD: Bone mineral apparent density
BMD: Bone mineral density
BMDD: Bone mineralization density distribution
BMI: Body mass index
CHAQ: Childhood Health Assessment Questionnaire
DXA: Dual-energy X-ray absorptiometry
ESR: Erythrocyte sedimentation rate
FWHMH: Full width at half-maximum height
JIA: Juvenile idiopathic arthritis
qBSE: Quantitative backscattered electron imaging
SLE: Systemic lupus erythematosis
